# Menstrual hygiene practice and associated factors among adolescent primary school females in Dale Woreda, Sidama, Ethiopia: a cross sectional study

**DOI:** 10.3389/frph.2025.1458132

**Published:** 2025-02-20

**Authors:** Amanuel Ayele, Meskerem G/Mariam, Hunachew Beyene, Alemu Tolcha, Dansamo Tediso, Teshale Shalamo, Teshale Belayneh

**Affiliations:** ^1^ Research and Community Service Directorate, Hawassa College of Health Sciences, Hawassa, Ethiopia; ^2^ Hawassa City Health Department, Alamura Health Center, Hawassa, Ethiopia; ^3^ School of Environmental Health, College of Medicine and Health Sciences, Hawassa University, Hawassa, Ethiopia; ^4^ Wonsho Woreda Health Office, Wonsho Woreda, Sidama Regional Health Bureau, Sidama, Ethiopia; ^5^ Department of Public Health, Hawassa College of Health Sciences, Hawassa, Ethiopia

**Keywords:** menstrual hygiene, practice, adolescents, associated factors, Sidama, Ethiopia

## Abstract

**Background:**

Despite poor menstrual hygiene practices exposing adolescent females to reproductive health problems, it has not been adequately studied in low-income countries. Due to a lack of proper information, the majority of teenage girls enter puberty without having prepared themselves. The aim of this study was to assess menstrual hygiene practice and associated factors among adolescent female students.

**Methods:**

An institution-based cross-sectional study design was employed from March 10 to April 20, 2022. A random sample of 608 adolescent female students was included in the study. A multistage sampling was applied to employee study participants. Data were collected using a structured questionnaire administered through an interview technique. A logistic regression model was used to analyze the data using version 26 SPSS statistical software. Descriptive statistics were used to summarize the data. An adjusted odds ratio with a 95% confidence interval and a corresponding *p*-value <0.05 was used to measure the strength of association.

**Result:**

The prevalence of poor menstrual hygiene practices among adolescent girls was 28.4%. Poor knowledge of adolescent girls [AOR = 2.64, 95% CI: 1.74, 4.02], mothers' educational level [AOR = 0.86, 95% CI: 0.79, 0.94], fathers' occupation of private employers and daily laborers [AOR = 3.1, 95% CI: 1.46, 6.69] and [AOR = 1.98, 95% CI: 1.03, 3.8], and having hand washing facilities [AOR = 0.51, 95% CI: 0.34, 0.77] were significantly associated with poor menstrual hygiene management practices among adolescent schoolgirls.

**Conclusion:**

In this study, we determined the level of poor menstrual hygiene practice. We identified factors significantly associated with menstrual hygiene practices. Interventions should focus on creating awareness among adolescent girls, providing menstrual hygiene management facilities, and improving the educational and occupational levels of parents.

## Introduction

A female reproductive system undergoes regular cyclic alterations that might be interpreted philosophically as periodic preparation for pregnancy and fertilization (1). The cycle is a menstrual cycle, and its most noticeable feature is the periodic vaginal bleeding that occurs with the shedding of the uterine mucosa. Menstruation begins throughout the adolescent era, which is characterized by significant physiological and emotional changes (1, 2). A significant number of schoolgirls in low-income countries face challenges with menstrual hygiene management practice facilities, which are worsened by ignorance of their menstrual needs by teachers and intimidation by boys and older girls, creating a stressful environment for schoolgirls (3–5). The prevalence of poor management of menstrual hygiene is significantly different among adolescent schoolgirls in different settings (6–9). Accordingly, worldwide, 335 million females attended schools where there was no access to water or soap (10). In the Middle East and Asia, up to 95% of girls claim that their menstruation is the reason they are missing school (11, 12). Around 13% to 75% of girls do not have access to clean sanitary materials and use low-quality products in low- and middle-income countries (6, 13, 14). In Sub-Saharan Africa, findings from a pooled analysis show that the practice of menstrual hygiene management among schoolgirls was 45% (15). Results from a pooled analysis in Ethiopia show that the menstrual hygiene practice was 52.69% (16). Other recent studies show that its practice ranges from 21% to 75% in Ethiopia (7, 8, 10, 17–22).

Prior evidence shows that several factors are influencing the practice of menstrual hygiene management (19, 23–29). These include: family size, cultural beliefs and societal norms, being able to afford menstrual sanitary products, place of residence, prior awareness on sexuality and access to a sanitary napkin, lack of knowledge, socio-economic and environmental constraints, the presence of a private latrine, and living with relatives (9, 15, 30–33). Age of the females, maternal educational level, frequency of discussion with mothers about menstruation, information source about menstruation, having knowledge about menstruation, academic level of the students, wealth indexed of the family, earning permanent pocket money, absence of storage container in the school, occupation of the parents, having infection around their vagina during menarche, place of residence, living with parents, were also identified as risk factors in Ethiopia (7, 10, 18, 19, 21, 34).

Ethiopia is a multicultural country that has more than 80 nations that follow different cultures and religions (35). The levels of menstrual hygiene practice significantly varied from region to region and place to place, as did its associated factors. There was an inconsistence of findings among studies that were previously done in Ethiopia (19, 21). Furthermore, there was a limited study that assessed the level of menstrual hygiene practice in the Sidama region. To our knowledge, there was no study done in Dale woreda. Therefore, the aim of this study was to assess the prevalence of menstrual hygiene practice and its associated factors among adolescents in the Dale Woreda School.

## Methods and materials

### Study setting

The study was done in Dale woreda, which is one of the administrative woredas of the Sidama national regional states, 311 km from Addis Ababa, the capital of Ethiopia. Dale is bordered from the south by Aleta Wendo and Cucko woreda, from the west by Lokka Abaya woreda, from the north by Shebedino woreda, and from the east by Wonsho woreda. Yirgalem is its capital town, and Woreda has 39 kebele. The estimated population of Woreda in 2014 EFY was 254,658 people, of whom 51% were female. The total estimated number of women belonging to the reproductive age group is 53,478. Dale woreda has 48 schools in total, and the total number of students attending grades 1–8 was 38,228; among them, 18,996 were female students. The total number of adolescent girls who are attending primary school at Dale woreda is 9,873.

### Study design and the source population

A school-based cross-sectional study was conducted in Dale district from March 20 to April 20, 2022. Adolescent girls attending the primary schools in the regular program and age group 10–17 were included in the study. Those girls whose menarche has not started, who have severe mental problems, refuse to participate, refuse to participate, and who were absent during data collection period were excluded from the study.

### Sample size determination and sampling procedure

A single population proportion formula was used to calculate sample size with the following assumptions: at 95% CI = 1.96, *P* = 60.3% proportion of poor menstrual hygiene practice in southern Ethiopia (36), d = margin of error taken as 5%. The sample size was calculated using the formula: *n* = (Z *α*/2)^2^ p (1-p)/d^2^, *n* = (1.96)^2^*(0.603) (1–0.603) (0.05)^2^, *n* = 368. By adding 10% of the non-response rate, the sample size becomes 405. Considering the design effect of 1.5 (to improve the efficiency or precision of an estimator), the final sample size was 608. A multi-stage sampling technique was applied to recruit the participants. First, Dale woreda was selected purposefully. From all schools found in Dale Woreda 10 schools were selected randomly. Each selected school received a proportional allocation of the total sample size. Then, adolescent girls from selected schools who fulfilled the inclusion criteria were enrolled in the study using a simple random sampling technique. To apply simple random sampling, a sampling frame was created by taking a list of adolescent girl students from each school.

### Main study outcome variable

Menstrual hygiene practice was the outcome variable in this study, which was assessed using a ten-item “yes” or “no” question. Each item's response was scored as “1” for correct answers and “0” for incorrect answers. The tool's total sum score ranges from 0 to 10. Girls with a total sum score less than the mean score are considered to have poor menstrual hygiene practices, while those who scored more than the mean score were considered to be good.

### Data collection tools and procedures

An interviewer-administered structured questionnaire was developed that addresses the objectives of the study after thorough reviewing relevant literature. A questionnaire was developed in English and translated to the local language (Sidaamu Afoo) by a language expert, then back to English to check its consistency. A five percent of the total sample size was pretested at another school to evaluate the validity of the instruments. The data were collected on socio-demographic, gynecologic, and knowledge-related characteristics. One-day training was given for both the data collectors and supervisor to explain the aim and content of the instrument. Data was collected by five female nurse professionals under supervision of one MSc. midwife professional. The data were checked for completeness and consistency on the spot during the data collection period.

### Data analysis

The completeness of the data swas checked, coded, and entered into Epi Data version 3.1. Then the data were exported and analyzed using Statistical Package for Social Sciences (SPSS) version 26. The data were summarized using the descriptive statistics. The binary logistic regression model was conducted to identify candidate variables for the final model, with a *p*-value of ≤0.25. The model's fitness was evaluated using the Hosmer-Lemeshow fitness of good test, which yielded a non-significant value, indicating the data fit reasonably well. An adjusted odds ratio (AOR) with a 95% confidence interval was employed to identify factors significantly associated with menstrual hygiene practices.

## Results

### Socio-demographic features of the participants

In our study, a total of 606 study subjects participated, with a response rate of 99.8%. Majority of the subjects (87.7%) were rural residents, with an average age of 14.9 years (SD ± 1.25 years). The majority of participants (94.4%) live with their parents. More than three-fourths of participants (79.9%) were protestant religion followers. Almost all (99.3%) subjects were from the Sidama ethnic group. More than two-thirds (68%) of participants attended grades 7 and above. More than four-fifths (87%) of participants mothers were housewives, and nearly three-fourths (71.1%) of participants fathers were farmers. Approximately three-fourths (74.5%) of participants receive money from their parents to buy sanitary pads. More than two third (71.0%) of participants had private latrine to change menstrual pads and nearly three fourth (72.3%) had got hand washing facility during changing pads (Table 1).

**Table 1:** Socio-demographic characteristic of adolescent female students in Dale Woreda, 2022; (*n* = 606).

Variables	Category	Frequency	Percentages
Residence	Urban	532	87.8
	Rural	74	12.2
Living with	Parents	572	94.4
	Relatives	29	4.8
	Others	5	0.8
Religion	Orthodox	33	5.4
	Protestant	484	79.9
	Muslim	26	4.3
	Others	63	10.4
Ethnicity	Sidama	602	99.3
	Gurage	4	0.7
Mothers educational level	Primary	575	94.9
	Secondary and above	31	5.1
Fathers educational level	Primary	457	75.4
	Secondary and above	149	24.6
Fathers’ occupation	Farmer	431	71.1
	Private employers	35	5.8
	Government employee	40	6.6
	Daily laborers	48	7.9
	Merchants	52	8.6
Mothers’ occupation	Housewife	518	85.5
	Farmers	9	1.5
	Private employers	5	0.8
	Government employers	1	0.2
	Merchants	73	12.0
Receive money from parents	Yes	450	74.3
	No	156	25.7
Availability of private latrine	Yes	430	71.0
	No	176	29.0
Availability of hand wash	Yes	438	72.3
	No	168	27.7

### Gynecologic characteristics of respondents

**Figure 1:**
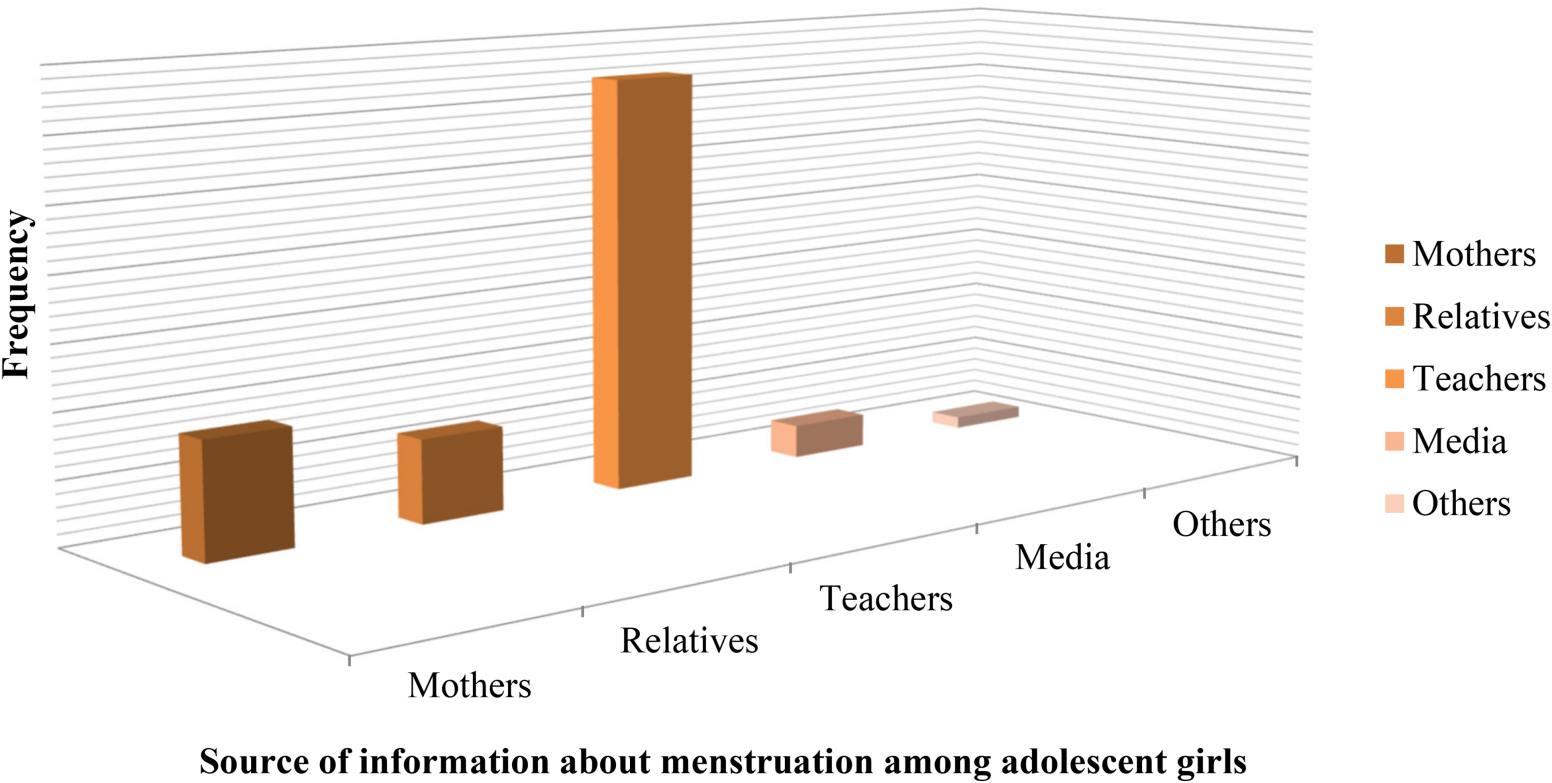
Source of information about menstruation for adolescent female students in Dale Woreda, 2022; (*n* = 606).

The mean age at menarche for participants was 12.85 (SD ± 1.05). More than four-fifths (84.5%) of participants got information about menstruation before the onset of menarche, of which nearly two-thirds (62.9%) got information regarding menstruation from their school teachers (Figure 1).

Majority (82.2%) of the respondents had regular menstruation and nearly half (45.4%) of them had a family history of dysmenorrhea. Majority (91.4%) of participants had a menstrual cycle which stays for 3–5 days duration (Table 2).

**Table 2:** Gynecologic characteristics of adolescent female students in Dale Woreda, 2022; (*n* = 606).

Variables	Responses	Frequency	Percentage
Having information about menstruation	Yes	512	84.5
	No	94	15.5
Family history of dysmenorrhea	Yes	275	45.4
	No	331	54.6
Regular menstruation	Yes	498	82.2
	No	108	17.8
Painful menstruation	Yes	210	34.7
	No	396	65.3
Duration of menstruation cycle	<3 days	40	6.6
	3–5 days	554	91.4
	>5 days	12	2.0

### Knowledge of participants toward menstruation

The majority (83.8%) of the participants knows that menstruation is a physiological process and three-fourths (75.1%) of participants believe that the cause of menstruation is hormonal. More than two-thirds (67.8%) of respondents know that the source of bleeding is the uterus. The majority (91.6%) of participants know about menstrual hygiene, and nearly all (95.4%) of the participants believe that menstruation has a foul smell. The majority (91.7%) of the participants knew that menstrual blood was not hygienic (Table 3).

**Table 3:** Knowledge of adolescent female students toward menstruation in Dale Woreda, 2022; (*n* = 606).

Variables	Response	Frequency	Percentage
What is menses	Physiological process	508	83.8
	Pathological process	3	0.5
	Curse from	2	0.3
	Don't know	93	15.3
Cause of menstruation	Hormones	455	75.1
	Curse of God	2	0.3
	Caused by diseases	1	0.2
	Don't know	148	24.4
Source of menstrual blood	Uterus	411	67.8
	Vagina	160	26.4
	Bladder	2	.3
	Abdomen	25	4.1
	Don't know	8	1.3
Heard about menarche before	Yes	543	89.6
	No	63	10.4
Know menstrual hygiene	Yes	555	91.6
	No	51	8.4
Know there is foul smelling	Yes	578	95.4
	No	28	4.6
Know menstrual blood is not hygienic	Yes	556	91.7
	No	50	8.3

**Figure 2:**
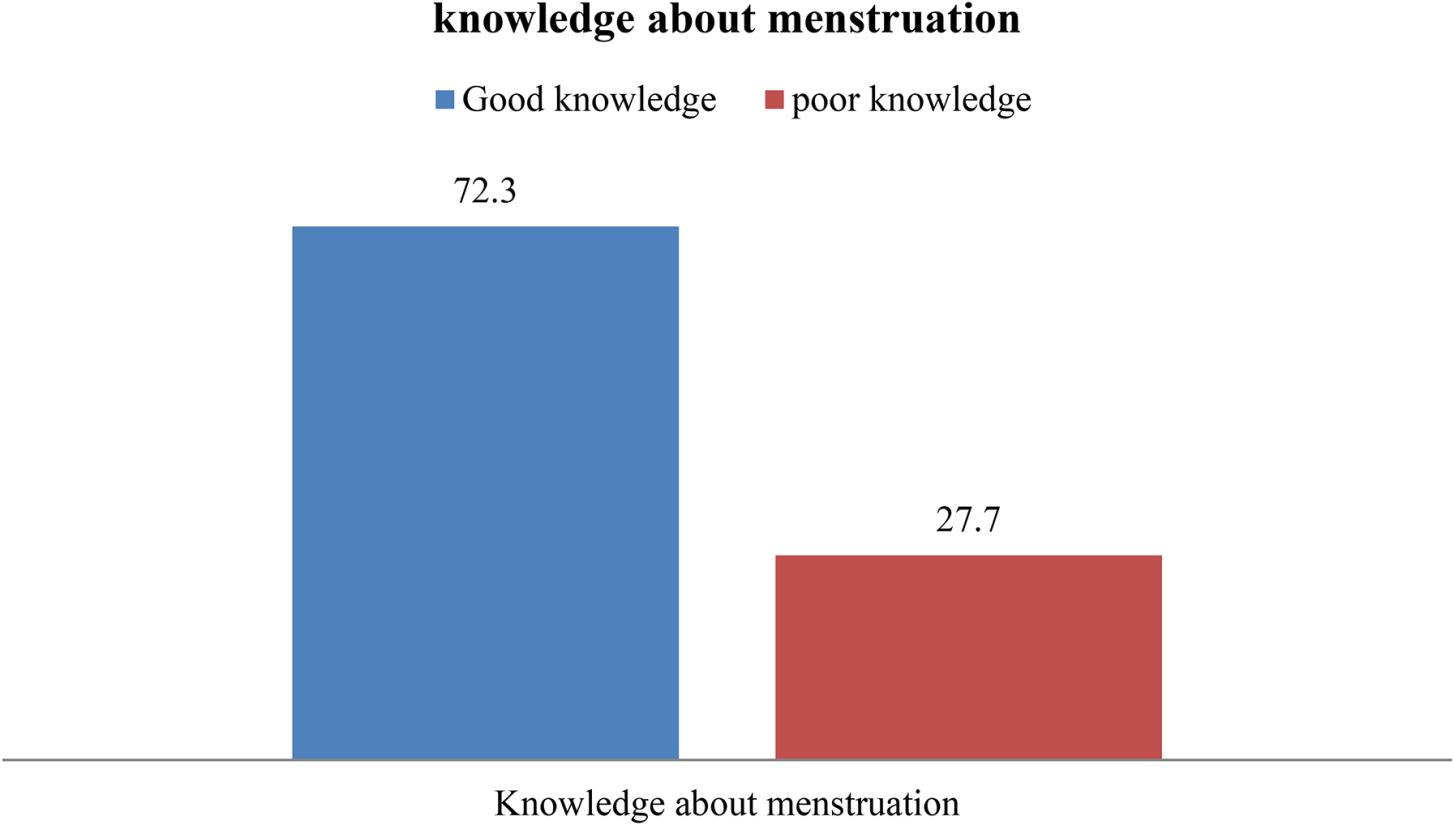
Knowledge of adolescent female students toward menstruation in Dale Woreda, 2022; (*n* = 606).

Regarding the overall knowledge level of the participant, nearly three fourth (72.3%) of respondents had a good knowledge about menstruation (Figure 2).

### Menstrual hygiene practice

Vast majority of the participants (96.0%) use absorbent pads during each menstruation cycle. Two-thirds of them (66.5%) use commercially available sanitary pads, and more than one-third (42.6%) change their pads three times per day. More than half (57.3%) of participants used clean cloth by washing with soap and water. Almost all (97.0%) of the respondents cleaned their external genitalia during menstruation, and most (94.4%) of them used soap and water. More than three-fourths (77.4%) of the participants discarded used pads by wrapping them, and most (91.9%) of them discarded used pads in dustbins. Nearly one third (32.3%) of the respondents took a bath daily during menstrual cycles (Table 4).

**Table 4:** Menstrual hygiene practices of adolescent female students in Dale Woreda, 2022; (*n* = 606).

Variables	Responses	Frequency	Percentages
Always use absorbent materials	Yes	582	96.0
	No	24	4.0
Always use commercially made sanitary Pad	Yes	403	66.5
	No	203	33.5
Change pads or Cloths more than three times a day	Yes	258	42.6
	No	348	57.4
Use clean clothes and wash with soap and water	Yes	347	57.3
	No	259	42.7
Commonly dry sanitary clothes with sunlight	Yes	347	57.3
	No	259	42.7
Clean my external genitalia	Yes	588	97.0
	No	18	3.0
Dispose the pads by wrapping	Yes	469	77.4
	No	137	22.6
Wash bathes daily with soap	Yes	196	32.3
	No	410	67.7
Clean external genitalia with water and soap	Yes	572	94.4
	No	34	5.6
Dispose used sanitary pads in dustbin/toilet	Yes	557	91.9
	No	49	8.1

**Table 5:** Factor associated with poor menstrual hygiene practice among adolescent female students in Dale Woreda; multivariable analysis (*n* = 606).

Variables	Response	Menstrual hygiene practice		COR (95% CI)	AOR (95% CI)	*p*-value
		Good	Poor			
Have information about menstruation	Yes	375	137	0.62 (0.39, 0.98)	1.3 (0.89, 2.3)	0.21
	No	59	35	1.0	1.0	
Regularity of menstruation	Yes	344	154	1.0	1.0	0.45
	No	90	18	2.24 (1.30, 3.80)	2.62 (1.51, 4.11)	
Earn money from parents	Yes	343	107	1.0	1.0	
	No	91	65	0.44 (0.30, 0.64)	0.97 (0.63, 1.50)	0.09
Mothers educational level	In grades			0.88 (0.81, 0.94)	0.86 (0.79, 0.94)	0.0001
Fathers’ occupation	Farmers	309	122	1.0	1.0	
	Private employer	21	14	1.70 (0.83, 3.43)	3.1 (1.46, 6.69)	0.0001
	Gov't employers	28	12	0.81 (0.37, 1.78)	1.16 (0.48, 2.66)	0.25
	Daily laborer	28	20	1.80 (0.98, 3.33)	1.98 (1.03, 3.80)	0.002
	Merchants	42	10	0.42 (0.16, 0.96)	0.55 (0.26, 1.30)	0.067
Age	≤13	58	11	2.3 (1.16, 4.4)	1.98 (0.91, 3.64)	0.075
	>13	376	161	1.0	1.0	
Knowledge about menstruation	Good	338	100	1.0	1.0	
	Poor	96	72	2.5 (1.74, 3.7)	2.64 (1.74, 4.02)	0.0001
Having hand washing facility	Yes	339	99	1.0	0.51 (0.34, 0.77)	0.0003
	No	95	73	2.63 (1.8, 3.8)	1.0	

**Figure 3:**
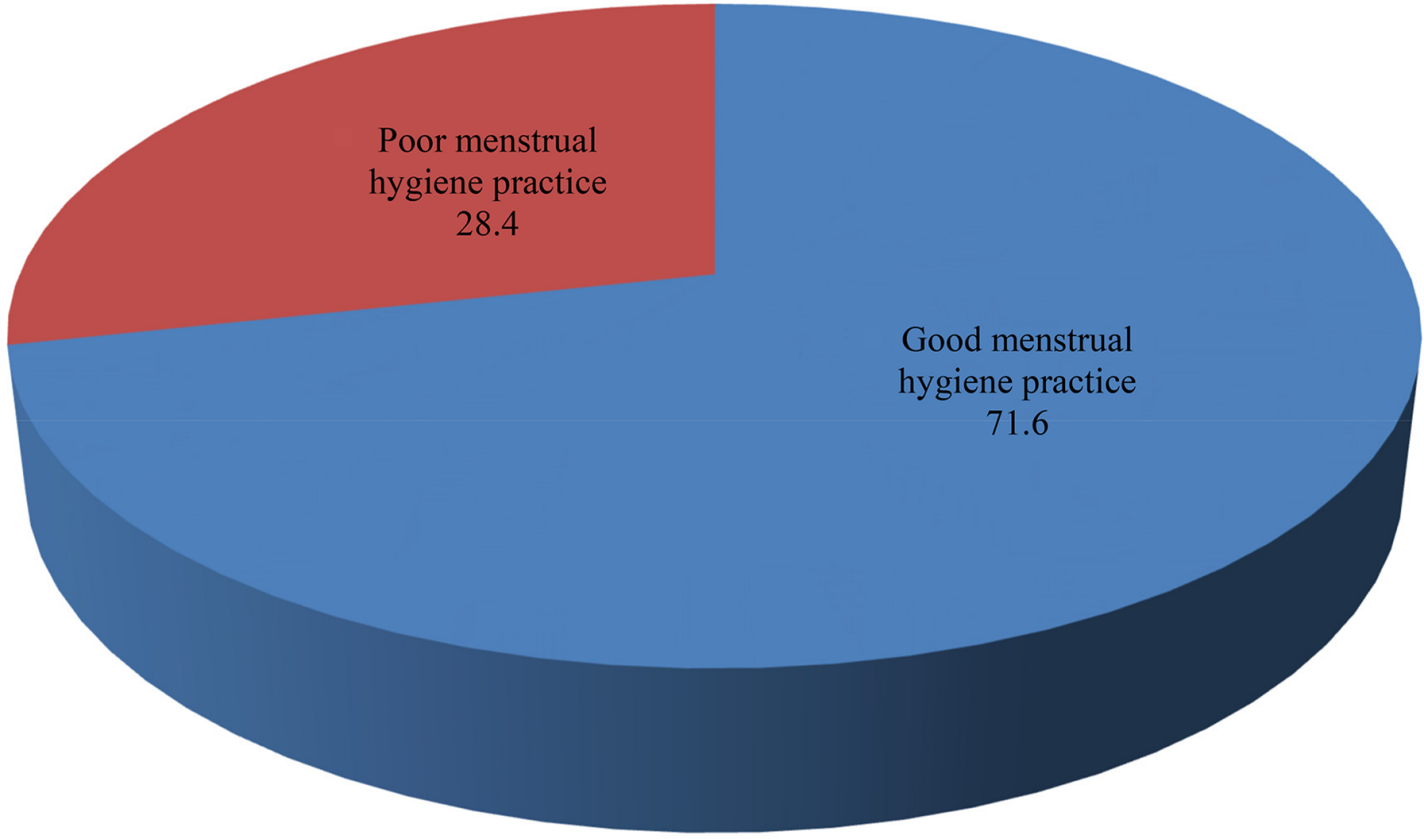
Menstrual hygiene management practices of adolescent female students in Dale Woreda, 2022; (*n* = 606).

The prevalence of poor menstrual hygiene practice was 28.4% (95% CI 25%–32%) which is more than one fourth (Figure 3).

### Factors associated with poor menstrual hygiene practice

Knowledge of adolescent girls toward menstruation, fathers' occupation, information about menstruation, regularity of menstruation cycle, earning money from parents, availability of hand washing facilities at the time of changing the menstrual pad, age of the female student, and mother's educational levels had *p*-values less than 0.25 with poor menstrual hygiene practice, in bivariate logistic regression.

After adjusting for potential confounders, knowledge of adolescent girls toward menstruation, fathers' occupation, availability of hand washing facilities at the time of changing menstrual pads, and mothers' educational level were associated significantly with poor menstrual hygiene practice among adolescent female students in dale woreda.

In our study, poor menstrual hygiene was significantly associated with the knowledge of adolescent girls. The odds of poor menstrual hygiene were three times [AOR = 2.64; 95% CI: 1.74, 4.02] higher among those who had poor knowledge regarding menstruation as compared to those who had good knowledge. The study found that menstrual hygiene was significantly associated with mothers' educational level. The odds of poor menstrual hygiene decreased by 14% [AOR = 0.86, 95% CI: 0.79, 0.94] as mothers' educational level increased by 1 grade compared to lower grades. In this study, fathers' occupations were significantly associated with poor menstrual hygiene practices. The odds of poor menstrual hygiene practice were three [AOR = 3.1; 95% CI: 1.46, 6.69] and two [AOR = 1.98; 95% CI: 1.03, 3.8] times higher among those whose parents work as private employers and daily laborers, respectively, as compared to farmers. The availability of hand washing facilities at the time of changing menstrual pads was related to a lower incidence of poor menstrual hygiene practices. The odds of poor menstrual hygiene practice were 49% [AOR = 0.51, 95% CI: 0.34, 0.77] less among those who had hand washing facilities at the time of changing menstrual pads as compared to their counterparts (Table 5).

## Discussion

The aim of this study was to assess menstrual hygiene practices and associated factors among adolescent female students in the Dale woreda. Accordingly, the prevalence of poor menstrual hygiene practice was 28.4% (95% CI: 25%–32%). Poor knowledge of adolescent girls concerning menstrual hygiene, mothers' educational level, fathers' occupation of private employers and daily laborers, and having hand-washing facilities were factors influencing menstrual hygiene practice among school girls. Its prevalence is still considerably high among schoolgirls due to the aforementioned and other contextual factors. To improve menstrual hygiene practice and keep dignity, gender-specific WASH facilities together with awareness creation activities should be implemented in the schools. In addition, schoolgirls require effective, safe, and free/affordable menstrual hygiene products to improve menstrual hygiene practice and academic performance or productivity.

This finding (28.4%) was consistent with a study conducted in Kenya, which reported that 28.8% of adolescent girls had poor menstrual hygiene practices (4). However, this finding was lower when compared with finding from studies conducted in, Dilla, Ethiopia, which showed poor menstrual hygiene management practices of 68.3% (36). Similarly, studies done in India and Ethiopia show a higher prevalence of poor menstrual hygiene management practices (46.4%) and 48.98%, respectively (37, 38). But the finding was higher than the finding from a study conducted in the Amhara region of Mehalmeda, Ethiopia, which showed poor menstrual hygiene management practices of 9.1% (39). The possible explanation for this discrepancy might be socio-demographic and cultural difference among participants. Additionally, poor menstrual hygiene practices, often a result of inadequate access to clean products, toilets, or hygiene education, can have significant health and social consequences (40–42). Good menstrual hygiene management involves safe product use, regular changing, proper disposal, and personal hygiene, all supported by education and resources (40, 43, 44). Studies consistently emphasize the importance of both individual behavior and systemic support in promoting menstrual health (45, 46). Good menstrual hygiene practices can prevent infections, discomfort, and other health complications, while poor menstrual hygiene practices can lead to various physical, emotional, and social challenges (47, 48).

This study found that poor menstrual hygiene was significantly associated with the knowledge level of adolescent girls. The odds of poor menstrual hygiene practice were 2.64 times higher among those who had poor knowledge regarding menstruation as compared to their counterparts. This finding was consistent with studies conducted in Ethiopia and Indonesia (8, 38, 39, 49), which shows, poor knowledge of girls was significantly associated with poor menstrual hygiene practice. A possible explanation for this might be that those adolescent female students who had good knowledge maintained their personal hygiene. Poor knowledge of menstrual hygiene among adolescent female students might make them prone to poor menstrual hygiene practices because they might have no idea what to do during and after menstruation.

Our study showed that having hand washing facilities at the time of changing the menstrual pad was significantly associated with poor menstrual hygiene practices. The odds of poor menstrual hygiene practices were 49% lower among those who had access to hand washing facilities as compared to those who had no access to hand washing facilities. This finding was in line with studies conducted in Kenya and the Amhara region, which showed access to hand washing facilities was associated with increased odds of good menstrual hygiene practices (4, 39). Water with hand washing facilities is an essential component of life to maintain hygiene and other day-to-day activities. Access to water and hand-washing facilities might help the adolescent female students maintain their menstrual hygiene and personal hygiene as well.

In this study, the occupation of a father was significantly associated with the menstrual hygiene practices of adolescent female students. The odds of poor menstrual hygiene practice were three and two times higher among adolescent female students whose parents' occupations were private employers and daily laborers, respectively, as compared to adolescent female students whose parent occupations were farmers. This finding was comparable to a study done in Ghana, which showed decreased odds of poor menstrual hygiene practices and being the daughter of a farmer as compared to other occupations (50). This might be due to the fact the fact that family socio-economic status influences girls' choice of menstrual care products and this may further explain the association between fathers’ occupation and the menstrual hygiene practices of the female students.

The current study found that mothers' educational level and menstrual hygiene practice were significantly associated. The odds of poor menstrual hygiene decreased by 14% when mothers educational level increased by 1 grade. This finding was consistent with different studies conducted in Ethiopia, Indonesia, and Nepal, which revealed that higher mother`s educational level was associated with decreased odds of poor menstrual hygiene practice (8, 49, 51, 52). A possible explanation for this could be that educated mothers might have a better awareness of menstrual hygiene practices, and thus, they could have an open discussion with their daughters about menstruation and/or more likely provide appropriate sanitary materials for them to maintain their menstrual hygiene.

### Limitation

The study was conducted on sensitive topic which might make it prone for social desirability bias. Furthermore, participants were adolescent female students aged from 10 to 17 years, they might forget thing easily, so this might cause information bias.

## Conclusion

This study was conducted to assess the menstrual hygiene management practices and associated factors of adolescent girls attending primary school in Dale woreda. The study found that poor menstrual hygiene management practice was significantly high. Poor knowledge of adolescent girls toward menstrual hygiene, lack of hand washing facilities at the time of changing menstrual pads, fathers' occupation being private employers and daily laborers, and mothers' educational level were factors significantly associated with poor menstrual hygiene practice in the study area among adolescent female students.

## Data Availability

The raw data supporting the conclusions of this article will be made available by the authors, without undue reservation.
